# Impact of Manufacturing Procedures on CAR T Cell Functionality

**DOI:** 10.3389/fimmu.2022.876339

**Published:** 2022-04-13

**Authors:** Norihiro Watanabe, Feiyan Mo, Mary Kathryn McKenna

**Affiliations:** ^1^ Center for Cell and Gene Therapy, Baylor College of Medicine, Texas Children’s Hospital and Houston Methodist Hospital, Houston, TX, United States; ^2^ Graduate Program in Translational Biology and Molecular Medicine, Baylor College of Medicine, Houston, TX, United States

**Keywords:** CAR T cell, *ex vivo* expansion, culture media, serum, cytokines, pharmacological inhibitor, manufacturing time, cryopreservation

## Abstract

The field of chimeric antigen receptor (CAR) modified T cell therapy has rapidly expanded in the past few decades. As of today, there are six CAR T cell products that have been approved by the FDA: KYMRIAH (tisagenlecleucel, CD19 CAR T cells), YESCARTA (axicabtagene ciloleucel, CD19 CAR T cells), TECARTUS (brexucabtagene autoleucel, CD19 CAR T cells), BREYANZI (lisocabtagene maraleucel, CD19 CAR T cells), ABECMA (idecabtagene vicleucel, BCMA CAR T cells) and CARVYKTI (ciltacabtagene autoleucel, BCMA CAR T cells). With this clinical success, CAR T cell therapy has become one of the most promising treatment options to combat cancers. Current research efforts focus on further potentiating its efficacy in non-responding patients and solid tumor settings. To achieve this, recent evidence suggested that, apart from developing next-generation CAR T cells with additional genetic modifications, *ex vivo* culture conditions could significantly impact CAR T cell functionality – an often overlooked aspect during clinical translation. In this review, we focus on the *ex vivo* manufacturing process for CAR T cells and discuss how it impacts CAR T cell function.

## Introduction

With the promising clinical success of CD19 CAR T cell therapy for B-cell lineage malignancies (1–4), there have been more and more publications focusing on ways to enhance CAR T cell function by complex genetic engineering (5–7). However, less attention has been paid to culture methods for the *ex vivo* maintenance of therapeutic T cells, a necessary step to generate CAR T cells for both preclinical research and clinical implementation, and their effects on the quality of cell products. The general procedure for manufacturing CAR T cell begins with isolation of peripheral blood mononuclear cells (PBMCs). Next, PBMCs or T cells that have been further enriched from PBMCs are stimulated with antibody-coated beads (e.g. Dynabeads) or plate-bound antibodies to induce T cell activation and then genetically modified using lentiviral vectors (8, 9), gamma-retroviral vectors (10, 11) or other delivery methods (12, 13) to express the cell surface CAR molecule. Subsequently, these engineered T cells are expanded in culture to reach the required cell numbers for either experimental testing or clinical treatment. Importantly, *ex vivo* culture conditions are completely different from the homeostatic environment *in vivo*, warranting detailed investigations on the impact of each manufacturing step on T cell quality. For instance, reagents used for *ex vivo* CAR T cell expansion, including media, sera, cytokines, and additional medium supplements, can collectively mount a significant impact on CAR T cell function. Additionally, the duration of CAR T cell expansion before cryopreservation can also affect the overall potency of CAR T cells. As the effects of different T cell enrichment/stimulation methods and gene delivery procedures have been extensively reviewed elsewhere (14), here we will focus on the impact of culture conditions, summarize and discuss how each component/step affects CAR T cell function.

## Impact of Culture Media

Selection of media is one of the first considerations for *ex vivo* CAR T cell expansion. Historically, RPMI-1640 medium has been widely used for T cell manufacturing. It is however unclear if this is the best choice for generating clinical-grade CAR T cells. Currently, there are a variety of media available in the market, all designed to support optimal T cell expansion. It is therefore important to compare and choose the right medium, with the first and foremost objective being sufficient T cell expansion that meets the required cell doses for treatment.

Sato et al. compared expansion of OKT3-stimulated PBMCs in different media (RPMI-1640, AIM-V and Optimizer) supplemented with low concentrations of autologous serum (1-8%) in the presence of IL-2 (175 IU/mL) (15). The 7-day fold expansion of T cells in each medium was 52.7 ± 13.1 (RPMI-1640), 25.9 ± 16.8 (AIM-V) and 55.5 ± 20.6 (Optimizer). However, fold expansion on day 12 was similar between Optimizer (1180 ± 51) and AIM-V (1110 ± 71) but lower in RPMI-1640 (not defined). Of note, serum concentration was 8% at the beginning of culture and was diluted every 2-3 days when new media were replenished, with the resulting serum concentration reaching 1% on day 7 and maintaining at that level. These results demonstrated that T cell expansion in RPMI-1640 is highly dependent on serum concentrations.

In another study, Lu et al. compared three serum-free media (SFM): Optimizer, X-VIVO15 and TexMACS, for *ex vivo* OKT3-stimulated T cell expansion in the presence of IL-2 (300 IU/mL) for 6 days (16). Using SFM is ideal for clinical manufacturing of CAR T cell products, since it prevents or reduces the risk of inconsistent CAR T cell functionality resulting from lot-to-lot variations of serum quality (17–19). Among the three SFM tested, Optimizer resulted in the greatest number of stimulated T cells. However, the overall cell expansion in all SFM conditions was significantly lower than that in the AIM-V medium supplemented with 5% human serum (HS), which was their institutional standard for CAR T cell manufacture.

Finally, Xu et al. performed a comprehensive medium comparison study evaluating the growth of anti-CD3/CD28-activated T cells in various medium formulations, either with or without serum addition (20). Here, they compared RPMI-1640 (+10%FBS), IMDM (+10%FBS), AIM-V (+10% human AB serum), Optimizer, X-VIVO 15, and StemSpan SFEM, all supplemented with exogenous IL-2 (1,000 IU/mL). Over the course of a 10-day *ex vivo* cell expansion period, the authors found that activated T cells maintained in the Optimizer medium achieved the highest T cell number. In addition, they characterized T cell memory phenotypes at the end of expansion, but did not observe any statistical difference among various culture conditions. It is important to point out that this finding is contradictory to the one reported by Lu et al. where AIM-V (+5% HS) outperformed Optimizer in terms of cell expansion. This could be in part due to the difference in the cytokine concentrations and/or sources of sera used by those two groups. Although future studies from independent groups with side-by-side comparisons are still required, these aforementioned three studies suggest that the choice of media has a significant impact on *ex vivo* expansion of OKT3- or OKT3/CD28-stimulated T cells used for CAR T cell generation.

In addition to cell expansion, an equally important goal is to generate CAR T cell products with the highest function possible. Medvec et al. compared CAR T cell function after growing them in different media (21) and reported that in the absence of serum supplementation, a chemically defined medium, 1B2H, supported *ex vivo* T cell proliferation to a similar level with that of the X-VIVO15 SFM, with selective expansion of T cells exhibiting a more differentiated phenotype (CCR7-CD27-). Anti-CD19 CAR T cells expanded in 1B2H showed potent *in vivo* anti-tumor activity with improved T cell persistence compared to CAR T cells expanded in X-VIVO15, even though CAR T cells maintained in X-VIVO15 contained slightly higher percentages of T cells with a less differentiated phenotype at the end of *ex vivo* expansion. However, these results conflict with a number of published studies demonstrating the importance of maintaining a less differentiated T cell phenotype for prolonged *in vivo* T cell persistence in adoptive T cell therapies (22–26). Although the authors did not elucidate the underlying mechanism of 1B2H-induced enhancement of *in vivo* anti-tumor activity, their data indicated that 1B2H might have the potential to improve expansion of CAR T cells generated from cancer patients whose T cells are prone to poor proliferation with a more differentiated phenotype. Notably, the authors also evaluated the effect of HS in 1B2H which will be discussed in the following section. Above results are summarized in **Table 1**.

**Table 1:** Impact of media on cell expansion.

Activation method	Cytokine	Medium	Serum	Expansion period	Cell expansion		Ref
Plate-bound OKT3 (3.3 μg/mL)	IL-2 (175 IU/mL)	RPMI-1640	autologous serum (8% gradually reduced to 1%)	7 and 12 days	52.7±13.1	Lowest (not specified)	(15)
		AIM-V			25.9±16.8	1110±71	
		Optimizer			55.5±20.6	1180±51	
Soluble OKT3 (50ng/mL)	IL-2 (300 IU/mL)	Optimizer	(–)	6 days	AIM-V(5%HS) > Optimizer > X-VIVO15 > TexMACS		(16)
		X-VIVO15					
		TexMACS					
		AIM-V	5% HS				
Dynabeads Human T-Activator CD3/CD28	IL-2 (1000 IU/mL)	RPMI-1640	10% FBS	10 days	272.0 ± 42.3 × 10^5^		(20)
		IMDM			344.0 ± 87.0 × 10^5^		
		AIM-V	10% AB serum		426.0 ± 46.6 × 10^5^	
		Optimizer	(–)		549.0 ± 82.7 × 10^5^	
		X-VIVO15			374.0 ± 98.0 × 10^5^	
		StemSpan SFEM			110.6 ± 23.4 × 10^5^	
Dynabeads Human T-Expander CD3/CD28	Not specified	RPMI-1640	(-)	15 days	1B2H ≈ X-VIVO 15 >>> AIM-V > RPMI-1640		(21)
		AIM-V					
		X-VIVO15		
		1B2H (+ glucose & galactose)		

## Impact of Sera and Serum Substitutes

While the cell therapy field is shifting towards SFM, most current clinical CAR T cell manufacturing protocols still utilize sera to support *ex vivo* T cell growth (16, 27). Currently, fetal bovine serum (FBS) and HS have been widely used for CAR T cell manufacture. Since the use of FBS may be immunogeneic and has the potential to transmit non-human pathogen(s), human-derived supplements are preferable for clinical application. In addition to HS, multiple serum substitutes derived from human blood have been tested in CAR T cell manufacture. For example, Ghassemi et al. investigated the effect of Physiologix XF (Phx), a concentrated extract from human transfusion grade whole blood fractions as a serum replacement, on CAR T cell expansion and function (28). They used different media, namely RPMI-1640, X-VIVO15 and Optimizer, and compared Phx (2%)-supplemented media with serum-containing media (10%FBS + RPMI1640, 5%HS + X-VIVO15, 5%HS + Optimizer). Phx-supplemented media exhibited comparable T cell expansion to serum-containing media, with the exception of the 10%FBS + RPMI condition that led to the greatest T cell expansion after 9-11 days of culture. The authors further analyzed the metabolites contained in Phx and HS and found modestly elevated levels of carnosine along with several monosaccharide derivatives in Phx compared to HS. Supplying carnosine to HS-containing media enhanced the expression of genes delivered by lentiviral vectors and shifted the metabolic profile of activated T cells from a glycolytic state to an oxidative one, which has been shown to correlate with superior anti-tumor function (29–32). Although the authors did not show the metabolic profile of CAR T cells expanded in Phx-supplemented Optimizer, in a mouse xenograft model of neuroblastoma, GD2 CAR T cells cultured under this condition exhibited more potent tumor control compared to T cells expanded in HS-containing Optimizer.

Our group has also explored the effect of three different types of sera: FBS, human AB serum and human platelet lysate (HPL), on CAR T cell function during *ex vivo* expansion (33). There was no difference in CAR T cell expansion when 10% of each serum was supplemented to the base medium (1:1 mixture of RMPI-1640 and Click’s medium), however, when serum concentrations were titrated down to 5% and 2.5%, FBS was unable to support CAR T cell expansion while similar levels of robust expansion were achieved across the HPL conditions regardless of concentrations. Strikingly, CAR T cells expanded in the HPL-supplemented medium maintained a large fraction of less differentiated T cells (naïve and central memory) compared to those cultured with other sera assessed by both cell surface phenotypes and gene expression signatures. As a result, HPL-cultured CAR T cells showed potent *in vivo* anti-tumor activity in both hematological (B cell leukemia treated with CD19 CAR) and solid tumor (pancreatic adenocarcinoma treated with PSCA CAR) models with prolonged T cell persistence. Another benefit of choosing HPL for CAR T cell manufacture is its lot-to-lot consistency, as demonstrated by Canestrari et al. in a study where they compared cytokine levels in ten different lots of HPL (34).

Because serum composition is very complex, it is hard to pinpoint which factor(s) in HPL is responsible for its superior performance. Nonetheless, we were able to show that transforming growth factor beta 1 (TGFβ1), which plays an important role in memory T cell pool formation (35, 36), is elevated in HPL compared to human AB serum by human proteomic analysis (34). Indeed, supplementing TGFβ1 into the FBS-containing medium greatly increased the percentage of CAR T cells with a less differentiated phenotype during *ex vivo* expansion, consistent with another independent report (37). However, not surprisingly, since TGFβ1 is a potent immunosuppressive cytokine, the anti-tumor effect of TGFβ1-exposed CAR T cells was strongly inhibited in both *in vitro* and *in vivo* experiments, suggesting that there are multiple cytokines/proteins in HPL contributing to enhanced CAR T cell function.

It is worth noting that although a number of publications, including aforementioned ones, have shown the benefit of serum supplementation, Medvec et al. reported negative effects resulting from serum addition under certain culture conditions (14), in a study described in the previous section. When comparing the effects of a chemically defined medium 1B2H and X-VIVO15 SFM, they found that CD19 CAR T cells showed significantly lower percentages of TNFα (+) and IL-2 (+) cells upon K562-CD19 stimulation when they were expanded in the presence of HS compared to those without HS. However, this was only observed when CAR T cells were generated from healthy donors rather than multiple myeloma patient samples. Despite this inferior cytokine secretion profile, 1B2H (serum-free)-expanded CAR T cells showed the most potent *in vivo* anti-tumor effects when compared to 1B2H (HS), X-VIVO15 (HS) and X-VIVO15 (serum-free) conditions. Although the underlying mechanism is still unknown, these results suggested that depending on the choice of medium, serum components might have an undesireable impact on CAR T cell function. Above results are summarized in **Table 2**.

**Table 2:** Impact of sera on CAR T cell function.

Serum	Medium	Cell expansion	Outcomes	Ref
10% FBS	RPMI-1640	Greatest expansion in 10% FBS+RPMI-1640 compared to other conditions	5% HS vs 2% Phx in Optimizer • enhanced lentiviral-mediated gene expression in Phx • enhanced GD2 CAR T cell activity *in vivo* in Phx	(28)
2% Phx				
5% HS	X-VIVO15			
2% Phx				
5% HS	Optimizer			
2% Phx				
10% FBS	RPMI-1640 + Click’s medium	Comparable in 10% serum conditions (lower expansion in FBS if % serum is reduced)	• HPL-maintained T cells had a less-differentiated phenotype *in vitro* • HPL-cultured CAR T cells exhibited enhanced proliferation upon *in vitro* antigen stimulation • HPL-expanded CD19 CAR and PSCA CAR T cells exhibited superior *in vivo* anti-tumor effect with prolonged T cell persistence	(33)
10% AB serum				
10% HPL				
(–)	X-VIVO 15	Comparable	CD19 CAR T cells cultured in 1B2H (no serum) showed better *in vivo* anti-tumor effect compared to the ones in 1B2H (5% HS)	(21)
5% HS				
(–)	1B2H (+ glucose & galactose)			
5% HS				

## Impact of Exogenous Cytokines

The addition of exogenous cytokines in cell culture promotes CAR T cell expansion and alters T cell phenotype and function. IL-2 is the most common cytokine used to expand CAR T cells including commercial products, such as KYMRIAH and YESCARTA (38, 39). Yet, preclinical studies utilizing other common gamma-chain (γc) cytokines such as IL-7, IL-15, and IL-21 have led to more effective immunotherapies and clinical investigations are underway (14, 40, 41). Therefore, the choice of cytokine supplementation during CAR T cell manufacturing must be considered to achieve enhanced T cell effector function and persistence.

In many current protocols, peripheral blood lymphocytes that are redirected to tumors with CARs are expanded in IL-2. IL-2 is primarily secreted from activated T cells and plays a role in T cell proliferation, differentiation, and contraction through activation induced cell death (42). IL-2 has been shown to promote both Th1 and Th2 effector T cell differentiation while inhibiting Th17 polarization. It also results in the expansion of regulatory T cells which express the high affinity IL-2 receptor and are known to limit inflammatory responses and impede anti-tumor activity (43). In a study comparing the expansion and efficacy of CD19 CAR T cells grown in IL-2 versus IL-7/15, two other homeostatic cytokines, IL-2-cultured CAR T cells (hereafter IL-2 CAR T cells) highly expressed the CAR molecule on day three post transduction and expanded 100 folds during two weeks of culture (44). IL-2 CAR T cells were robust in killing tumor cells both *in vitro* and *in vivo*, but overtime CAR T cells expanded in IL-7/15 outperformed IL-2 CAR T cells with increased expansion and improved persistence. Furthermore, IL-2 CAR T cells contained more regulatory T cells and expressed higher levels of PD-1 after multiple rounds of antigen stimulation, leading the authors to suggest that IL-2 CAR T cells are more exhausted compared to IL-7/15-expanded ones. Similar results were also found by Xu and colleagues that among CAR T cells expanded with different common γc cytokines, IL-2-exposed CAR T cells exhibited the poorest anti-tumor function (45). This particular study comprehensively measured the effect of single cytokine supplement on an anti-Folate receptor alpha-CAR and determined that addition of IL-2, IL-7, or IL-15 resulted in the greatest fold expansion compared to other conditions including no cytokine, IL-18, and IL-21. However, IL-21-exposed CAR T cells exhibited the greatest expansion of less differentiated CAR T cells defined by the CD62L+CCR7+CD27+CD28+ phenotype and therefore prolonged persistence in *in vivo* models. The authors suggested that while IL-2 had been widely used in the generation of clinical-grade CAR T cells, it might not be the best condition, but rather IL-7- and IL-15-expanded CAR T cells exhibited better properties (expansion, cytotoxicity, cytokine secretion) before *in vivo* infusion and IL-15- and IL-21-cultured CAR T cells might be best suited for optimal *in vivo* activity (45).

IL-21 is also a member of the common γc cytokine family and has been shown to enrich a less differentiated phenotype of T cells (46). In combination with IL-2, IL-21 supplemented to CAR T cell culture media was found to increase CAR T cell proliferation, promote outgrowth of naïve and memory T cells, improve anti-tumor function and also increase the expression of CAR molecules on the surface of T cells transfected with the Sleeping Beauty transposon (47). The improved CAR expression with IL-21 supplement after lentiviral transduction was also observed in studies by Du et al., through dampened IFNγ expression (48). While IL-21 alone did not result in robust CAR T cell proliferation (45), when used in combination with other cytokines such as IL-7 and IL-15, IL-21 improved CAR T cell effector function (48).

With the discovery that T cells exhibiting a less differentiated phenotype, such as stem-cell like memory cells (T_SCM_), are correlated with improved clinical outcomes, many studies have worked to maintain this population in CAR T cell products (49). In an effort to continue the use of IL-2, Kaartinen and colleagues titrated the dose of IL-2 for CD19 CAR T cell expansion and found that low doses of IL-2 (5 U/ml) resulted in less differentiated T cells but at the cost of reduced expansion, similar to that of no cytokine supplementation, and less effector function (50). The authors did note that if *in vitro* expanded T_SCM_ cells exhibited similar homing features to their physiological counterparts, then those cells might not have a strong capacity to enter the periphery and reach the tumor sites, and thus might not be the best product for solid tumor treatment. However, studies to preserve minimally differentiated T cells in *ex vivo* expansion are of current interest due to their potentially enhanced anti-tumor activity *via* increased persistence. Ultimately the combination of cytokines such as IL-7 and IL-15 have shown promise to preserve CD45RA+CCR7+ and CD45RA-CCR7+ T cells with improved proliferation after antigen exposure (42, 51). Above results are summarized in **Table 3**.

**Table 3:** Impact of exogenous cytokines on CAR T cell expansion and function.

CAR	Cytokine		Expansion	Phenotype	Notes	Ref
19BBz	IL-2 (200U/mL)		100-fold (2 weeks)	Promoted CD8+ Effector Memory (CD45RA-CD62L-, CD45RA+CD62L-)	- Treg expansion	(44)
	IL-7 (10ng/mL) +IL-15 (10ng/mL)		>200-fold (2 weeks)	Promoted CD8+ naïve (CD45RA+CD62L+) & central memory (CD45RA-CD62L+)	- ↓ apoptosis - ↑ engraftment - ↑ anti-tumor activity	
C4-27z & CD19-27z	IL-2 (10ng/mL)		150-fold (2 weeks)	Mature Effectors (CD45RA-CD62L- & CD45RA+CD62L-)	- ↓ apoptosis, - ↑TNFα	(45)
	IL-7 (10ng/mL)		150-fold (2 weeks)	Highest T_SCM_ population (CD45RA+CD62L+CD95+)	- ↓ apoptosis - ↑ proliferation capacity	
	IL-15 (10ng/mL)		150-fold (2 weeks)	Promote T_CM_ (CD45RA-CD62L+)	- ↓ apoptosis - ↑ anti-tumor activity *in vivo*
	IL-18 (10ng/mL)		30-fold (2 weeks)	Phenotype similar to no cytokine control
	IL-21 (10ng/mL)		50-fold (2 weeks)	Increased CD62L+	- ↑ Granzyme - ↑ *in vivo* T cell persistence - Preserved CAR expression
CD19-CD28z	IL-2	0 U/mL	11-fold (10 days)	Early memory T cells (T_SCM_) CD95+CD45RO−CD45RA+CD27+ present with low IL-2 and short expansion time	- More IL-2 decreased early memory T cells	(50)
		5 U/mL	12-fold (10 days)			
		20 U/mL	39-fold (10 days)		
		100 U/mL	60-fold (10 days)		
		300 U/mL	62-fold (10 days)		
CD19RCD28	IL-2 (50U/mL) +IL-21 (30ng/mL)		39.2-fold (14 days)	CD8+ memory and naïve cells	- ↑ CAR expression overtime - ↑ antitumor activity compared to IL-2 alone	(47)
Her.2 BBz	IL-2 (150U/mL)		Approx.100-fold (12 days)	IL-21 promoted naïve like and T_CM_	-IL-21 ↑CAR expression by reducing IFNγ - IL-15 + IL-21 best combination for expansion and effector function	(48)
	IL-7 (10ng/mL)		Approx.20-fold (12 days)			
	IL-15 (10ng/mL)		Approx.70-fold (12 days)			
	IL-2 (150U/mL) +IL-21 (20ng/mL)		>100-fold (12 days)			
	IL-7 (10ng/mL) +IL-15 (10ng/mL)		Approx.70-fold (12 days)			
	IL-7 (10ng/mL) +IL-21 (20ng/mL)		Approx.30-fold (12 days)			
	IL-15 (10ng/mL) +IL-21 (20ng/mL)		>100-fold (12 days)			
	IL-7 (10ng/mL) +IL-15 (10ng/mL) +IL-21 (20ng/mL)		>100-fold (12 days)			

Interestingly, besides the common γc cytokine family and IL-18, *ex vivo* expanded CAR T cells in the presence of TGFβ1 has also been shown to promote central memory T cell accumulation and BCMA-targeting CAR T cells exhibited improved anti-tumor activity when exposed to TGFβ1 (37).

## Impact of Pharmacological Inhibitors

Standard *ex vivo* expansion procedures often inevitably accelerate terminal differentiation and senescence of T cells, since initial TCR stimulation, CAR tonic signaling, and cytokine signaling can all lead to activation of signaling transduction pathways that drive effector T cell differentiation. In addition, as most cancer patients have undergone multiple rounds of pre-treatment, their T cells often exhibit an exhausted and/or senescent phenotype, resulting in poor expansion and functionality of their CAR T cell products. Therefore, multiple studies supplemented T cell culture with pharmacological inhibitors that specifically suppressing cellular programs that drive T cell terminal differentiation, exhaustion, and/or senescence, in order to reinvigorate patients’ T cells and improve CAR T cell fitness during manufacture (**Table 4**).

**Table 4:** Summarization of studies on pharmacological inhibitors.

Pharmacological inhibitors	Cellular targets	CAR specificity	Ref
Akt inhibitor VIII (aka Akti-1/2; PubChem Compound Identification: 10196499)	AKT	CD19	(52, 53)
LY294002	PI3K	CD33	(54)
bb007	PI3K	BCMA	(55)
Idelalisib (aka CAL-101)	PI3Kδ	CD19	(56)
Idelalisib (aka CAL-101)	PI3Kδ	Mesothelin	(57, 58)
Umbralisib (aka TGR-1202)	PI3Kδ	Mesothelin	(57)
Eganelisib (aka IPI-549)	PI3Kγ	Mesothelin	(57)
Duvelisib	PI3Kδ/PI3Kγ	CD19	(59)
Ibrutinib	ITK	CD19	(60)
TWS119	GSK-3β	CD19	(61)

As integrated signals from CD3ζ, costimulatory molecules and cytokine receptors lead to activation of a vast signaling transduction network in T cells, the majority of studies targeted critical components of a chosen signaling pathway, in order to selectively block pathways that mediate effector T cell differentiation while sparing pathways that contribute to T cell activation, proliferation, and memory formation. The most extensively explored pathway to date is PI3K/AKT/mTOR, a pathway known to facilitate T cell proliferation and effector differentiation through promoting glycolytic metabolism and functional suppression of the transcription factor FOXO1 (60, 62). Two independent groups reported that compared to conventionally grown CD19 CAR T cells, the addition of AKT inhibitors during manufacture resulted in enrichment of CAR T cells with a CD62L-expressing central memory (T_CM_) phenotype and enhanced anti-tumor efficacy in different xenograft models, without compromising cell yields (62, 63). Multiple other studies have targeted PI3K, a kinase upstream of AKT, and demonstrated that PI3K blockade helped a variety of CAR T cells maintain a less differentiated phenotype with enhanced *in vivo* persistence and anti-tumor efficacy (52–57). Of note, one study compared the effect of blocking AKT versus PI3Kδ (a subset of PI3Ks) using mesothelin-specific CAR T cells and found that PI3Kδ inhibition upregulated the stem cell memory transcription factor TCF7 more than AKT inhibition, translating to better *in vivo* anti-tumor efficacy (56). Another study compared the efficacy of inhibitors specific to different subsets of PI3Ks and found that PI3Kδ suppression resulted in better CAR T cell functionality compared to PI3Kγ blockade *in vitro* (55). Importantly, two studies also demonstrated the feasibility of utilizing PI3K blockade to generate CD19 CAR T cells with superior anti-tumor activity in xenograft models of chronic lymphocytic leukemia (CLL) and acute lymphocytic leukemia (ALL), using T cells derived from CLL patients (54, 57).

Apart from targeting the PI3K/AKT/mTOR pathway components, several studies took advantage of other signaling pathways involved in T cell activation. One such study used ibrutinib to inhibit interleukin-2-inducible T-cell kinase (ITK) signaling that is involved in T cell differentiation (58). This approach improved the overall quality of CD19 CAR T cells generated from CLL patients, in that the ibrutinib-treated CAR T cells expressed lower percentages of exhaustion markers including PD-1, TIM-3 and LAG-3, and had elevated TNFα and IFNγ production following target stimulation *in vitro* (58). Another group used sorted CD8+CD62L+CD45RA+ naïve precursor T cells as the starting material, genetically engineered and expanded them in the presence of a mixture of IL-7, IL-21 and glycogen synthase kinase 3β (GSK-3β) inhibitor TWS119 (59). As GSK-3β destabilizes β-catenin (61), an activator of transcription factors (e.g., LEF/TCF) that facilitate expression of genes responsible for memory T cell differentiation, addition of the GSK-3β inhibitor along with IL-7 and IL-21 enriched for CD19 CAR-expressing CD45RO-CCR7+CD45RA+CD62L+CD27+CD95+ T_SCM_ that showed enhanced metabolic fitness and long-lasting anti-tumor activity in a leukemia xenograft model. A third study identified that inhibition of p38 kinase, a key driver of T cell senescence (64, 65), led to an increased percentage of CD62L^+^ human CD19 CAR T cells with elevated IFNγ production following *in vitro* stimulation (66). They also showed that murine CD19 CAR T cells cultured in the presence of the p38 inhibitor had augmented anti-tumor activity *in vivo* (66). Last but not least, it has been reported that reversible yet complete blockade of CAR tonic signaling during manufacture using dasatinib (67, 68), a Src-family kinase inhibitor suppressing the function of LCK/FYN (69) that transmits CAR activation signals from CD3ζ to downstream Syk-family kinases, resulted in GD2 CAR T cells enriched for the CD62L+CD45RO+ T_CM_ subset, with lower expression of exhaustion markers including PD-1, TIM3, and LAG-3, as well as augmented *in vivo* function in a xenograft tumor model, compared to non-treated CAR T cells (70).

Besides signaling cascade blockade, other efforts to maintain minimally differentiated CAR T cells include epigenetic and metabolic interventions. One example is the a bromodomain and extra-terminal motif (BET) protein inhibitor JQ-1, which enabled enrichment of human CD19 CAR T cells with T_SCM_ and T_CM_ phenotypes as well as superior *in vivo* persistence and anti-tumor effects (71). Notably, *ex vivo* treatment of JQ-1 also reinvigorated exhausted and dysfunctional CD19 CAR T cells sourced from non-responding CLL patients (72). Mechanistically, BET proteins represent a protein family responsible for epigenetic marker recognition and transcriptional factor recruitment. Suppression of the BET protein BRD4 downregulates BATF gene expression in CD8^+^ T cells, thereby inhibiting T cell differentiation into the effector memory phenotype (71). Blocking BRD4 also leads to upregulation of CAR transgene expression and methylcytosine dioxygenase TET2 gene (73) downregulation, ultimately improving functionality of CAR T cells derived from non-responding CLL patients (72). In terms of metabolic regulation, one study used avasimibe to inhibit the function of ACAT1, a key cholesterol esterification enzyme that reduces the plasma membrane cholesterol level of CD8+ T cells, thereby decreasing the level of TCR clustering and signaling (74). Blocking ACAT1 resulted in enrichment of CD8+ CD19 CAR T cells with enhanced *in vitro* cytotoxicity (75).

In addition to enriching T cells with a desirable memory phenotype, recent development also utilized pharmacological inhibitors to maintain CAR T cells with other preferred functionality. Nian et al. (76) showed that tonic signaling of EpCAM-specific CAR T cells during *ex vivo* expansion resulted in hyperactivation of mTORC1, which downregulated CXCR4 and impaired the ability of CAR T cells to migrate to bone marrow. By using rapamycin to attenuate mTORC1 signaling during *ex vivo* expansion, the treated CAR T cells, compared to untreated CAR T cells, had elevated CXCR4 expression, enhanced bone marrow infiltration capacity, and augmented cytotoxicity against bone marrow resident leukemic cells in various xenograft models of human acute myeloid leukemia (AML). In addition, they also observed similar functional improvement using a CD33-specific CAR, indicating that this strategy might be generally applicable with different CAR constructs.

## Impact of Culture Period

Currently, T cells for generating CAR T cell products are sourced from patients themselves. The total manufacturing time varies depending on how much starting material (PBMCs or isolated T cells) is available and how fast patient-derived T cells grow. In some cases, patients failed to respond to treatment due to rapid disease progression during the lengthy manufacturing period. Therefore, much effort is being made to reduce the manufacturing time. Moreover, studies also suggested that a shortened *ex vivo* culture period correlated with improved CAR T cell functionality. Ghassemi et al. (77) compared function of CD19 CAR T cells harvested after short- (day 3 or 5) and long-term (day 9) culture. The authors showed that short-manufactured CAR T cells exhibited more robust tumor control compared to long-manufactured ones in a mouse xenograft model. Other groups have observed comparable results in similar xenograft mouse models (78, 79) as well as clinical trials (78, 80). In reality, due to prior lymphodepleting cancer treatment, the short-term *ex vivo* expansion protocol may not be feasible for patients with reduced T cell counts and activity. Nevertheless, these results suggested that circumstances permitting, shortened manufacturing time should be opted for. It is however worth noting that although the above studies mentioned maintenance of a less-differentiated T cell phenotype in short-manufactured CAR T cells as a reason for functional enhancement, the surface makers they used to define memory populations including CCR7 and CD45RO will appear artificially high shortly after antigen stimulation, including *in vitro* CD3 stimulation (81–85). Therefore, it requires extra caution when interpreting memory phenotype data from highly activated CAR T cells that are produced in a short manufacture period.

## Impact of Cryopreservation

At the end of the manufacturing process, CAR T cells are often cryopreserved, allowing sufficient time to complete quality control tests and flexible scheduling for infusion into patients. As cryopreservation may affect the viability and functionality of T cells, several groups have looked into its effect on CAR T cell products. Lee et al. demonstrated that there was no difference between fresh and frozen/thawed CD20 CAR T cells in terms of phenotype, cytokine secretion and *in vivo* cytotoxicity (86). Of note, they used the Cryostor medium to freeze CAR T cells, stored in liquid nitrogen overnight, and then thawed the next day for experimental use. A similar preclinical observation was reported by Xu et al. where *in vivo* functionality of BCMA CAR T cells between fresh versus one-month cryopreservation (10%DMSO + 90% FBS) groups was comparable, with only a slight decrease of cytokine levels produced from frozen/thawed CAR T cells (87).

To investigate the effect of cryopreservation on CAR T cell products used in the clinic, Panch et al. (88) retrospectively evaluated data from a total of 158 frozen/thawed autologous CAR T cell lines and PBMCs that are used as starting material for CAR T cell generation (freezing medium containing 5% DMSO and 6% pentastarch with 4% human serum albumin) across 6 single-center clinical trials. Overall, cryopreservation procedure did not affect clinical outcomes. In addition to patient samples, they also looked into frozen/thawed CAR T cells generated from healthy donors and demonstrated that these cells had early apoptotic cell surface markers and activation of apoptotic pathways, mitochondrial dysfunction, and cell cycle damage pathways (88). In another retrospective clinical study, Su et al. reported similar results where fresh and cryopreserved (Cryostor-CS10) CD19 CAR T cells produced comparable clinical outcomes (89). In contrast, Shah et al. observed that peak CAR T cell expansion levels and the overall response rate (ORR) were improved in patients who received fresh CD20/CD19 tandem bispecific CAR T cells compared to cryopreserved ones, indicating potential advantages of fresh products over frozen/thawed ones (90). Given the limited number of reports available, more future studies are needed to determine the impact of cryopreservation.

## Other Factors That May Impact CAR T Cell Function

As described above, *ex vivo* CAR T cell manufacture involves multiple, albeit straightforward, steps and variable factors. Beyond those reviewed above, there are other factors that may affect CAR T cell function. During Current Good Manufacturing Practice (CGMP) manufacture, CAR T cells can be expanded in culture plates, flasks, bags or much larger culture vessels such as G-Rex devices and rocking bioreactors (e.g. WAVE bioreactors) to simplify the process. Furthermore, CliniMACS Prodigy system provides a fully-automated manufacture system to generate clinical-grade CAR T cells. Advantages and disadvantages of these devices have been summarized in detail by another review (91). Validation studies of CAR T cell manufacture in each system (92–99) to date mainly focused on cell yields. One should keep in mind that it is difficult to compare the performance of different devices due to their distinct characteristics, such as volume of medium added, being a closed or open (semi-open) system and “static vs moving” culture. Those factors further dictate the optimal cell seeding density, efficacy of gas exchange and degree of pH alteration in each device. Although a simplified and large scale closed system is preferable for clinical CAR T cell manufacture due to its consistency in product quality and reduced complexity as well as risk of contamination, most preclinical studies utilize culture plates and flasks. Therefore, it is important to consider the effect of different culture devices for CAR T cell manufacture. During clinical translation, standard operating procedures need to be tailored to individual devices in order to maximize CAR T cell potency.

## Discussion

Although extensive efforts have been spent on inventing genetic engineering methods to enhance CAR T cell potency, fewer studies to date focused on the optimization of *ex vivo* cell expansion conditions. However, as discussed in the current review, the choice of reagents for *ex vivo* CAR T cell expansion can drastically affect the quality of therapeutic T cells, emphasizing the need for more detailed investigations. In general, most studies have found that culture methods leading to preservation of less differentiated, less exhausted, and/or less glycolytic CAR T cells during *ex vivo* expansion yielded T cell products with higher anti-tumor potency *in vivo*. This is consistent with prior observations that enhanced glycolytic metabolism impaired long-term memory formation of CD8+ CAR T cells (29, 31) and that CAR T cells generated from T_N_ and T_CM_ populations showed superior *in vivo* performance compared to CAR T cells derived from T_EM_ cells (22–26). Further, clinical evidence indicated that CAR T cells in complete-responding patients were enriched in memory-related genes while those from non-responders upregulated transcriptional pathways associated with effector differentiation, glycolysis, exhaustion and apoptosis (30). These data suggested that CAR T cells with long-lived memory phenotypes and enhanced metabolic fitness are desirable for clinical use.

During clinical translation, while it might be pragmatically difficult to standardize culture conditions for CAR T cells across different institutions, careful considerations must be given to the manufacturing process design, in order to maximize the potency of each final product. It is important to note that our knowledge about the effect of manufacturing methods remains insufficient, in that the number of studies investigating this aspect is limited and that many of these efforts relied on CAR T cell expansion (product quantity) as the sole readout, while overlooking the importance of CAR T cell functionality (product quality). To fill this gap of knowledge, future studies, especially clinical trials, will need to systemically evaluate the impact of different *ex vivo* expansion methods during each manufacturing step on both the quantity and quality of therapeutic T cells. Furthermore, new types of media, sera and serum substitutes, as well as other reagents are expected to be developed in the future, which will provide combinatorial and synergistic effects to boost CAR T cell function. Combining the optimal manufacturing procedure with additional innovative genetic engineering approaches will allow us to achieve our ultimate goal of developing an effective CAR T cell therapy for cancer patients.

## Author Contributions

FM drafted the impact of pharmacological inhibitor section. MM drafted the impact of exogenous cytokine section. NW drafted all remaining sections. All coauthors reviewed and edited the final manuscript. All authors contributed to the article and approved the submitted version.

## Funding

This work was supported by the National Heart, Lung, and Blood Institute 5T32HL092332-17 and the National Cancer Institute F99CA253757. Cancer Prevention and Research Institute of Texas HIHR RP210158.

## Conflict of Interest

NW received research funding from Sexton Biotechnologies for HPL-related studies.

The remaining authors declare that the research was conducted in the absence of any commercial or financial relationships that could be construed as a potential conflict of interest.

## Publisher’s Note

All claims expressed in this article are solely those of the authors and do not necessarily represent those of their affiliated organizations, or those of the publisher, the editors and the reviewers. Any product that may be evaluated in this article, or claim that may be made by its manufacturer, is not guaranteed or endorsed by the publisher.
